# Pharmacometabolomics via real-time breath analysis captures metabotypes of asthmatic children associated with salbutamol responsiveness

**DOI:** 10.1016/j.isci.2024.111446

**Published:** 2024-11-20

**Authors:** Jiafa Zeng, Jakob Usemann, Kapil Dev Singh, Anja Jochmann, Daniel Trachsel, Urs Frey, Pablo Sinues

**Affiliations:** 1 Department of Biomedical Engineering, University of Basel, 4123 Allschwil, Switzerland; 2 University Children’s Hospital Basel UKBB, University of Basel, 4056 Basel, Switzerland

**Keywords:** Health sciences, Medicine, Medical specialty, Internal medicine, Respiratory medicine, Diagnostics

## Abstract

Asthma is a widespread respiratory disease affecting millions of children. Salbutamol is a well-established bronchodilator available to treat asthma. However, response to bronchodilators is very heterogeneous, particularly in children. Pharmacometabolomics via exhaled breath analysis holds promise for patient stratification. Here, we integrate a real-time breath analysis platform in the workflow of an outpatient clinic to provide a detailed metabolic snapshot of patients with asthma undergoing standard clinical evaluations. We observed significant metabolic changes associated with salbutamol inhalation within ∼1 h. Our data supports the hypothesis that sphingolipid metabolism and arginine biosynthesis mediate the bronchodilator effect of salbutamol. Clustering analysis of 30 metabolites associated with these pathways revealed characteristic metabotypes related to clinical phenotypes of poor bronchodilator responsiveness. We propose that such a metabolic fingerprinting approach may be of utility in clinical practice to quantify response to inhaled medications or asthma outcomes.

## Highlights

• Pharmacometabolomics via breath analysis was integrated into an outpatient clinic
• Salbutamol-induced metabolic changes were identified in breath samples
• Sphingolipid metabolism and arginine biosynthesis pathways showed enrichment
• Breath analysis revealed metabotypes linked to salbutamol responsiveness

## Graphical abstract

**Figure:**
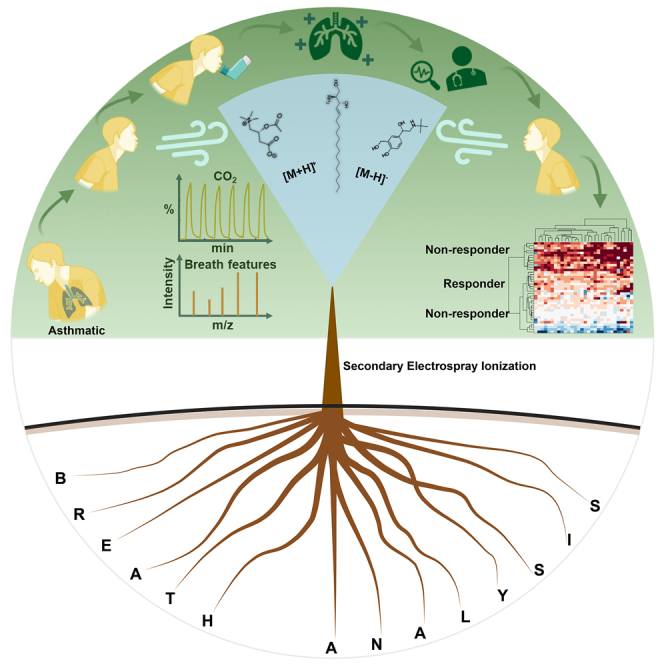

## Introduction

Asthma is a common and heterogeneous chronic respiratory disorder impacting millions of children.^1^^,^^2^^,^^3^ Among the commonly employed medications for asthma management, salbutamol is one of the most widely used. It is classified by the World Health Organization as one of the most effective and safest medicines indispensable for healthcare systems.^4^ The inhaled form of the medication typically onsets within approximately 15 min, with the duration of effect lasting between 2 and 6 h and peak plasma concentrations taking place after around 2 h.^5^ Salbutamol is a short-acting β_2_-agonist (SABA).^6^ Its mechanism of action entails adenylate cyclase activation, which boosts the synthesis of cyclic adenosine monophosphate (cAMP) within cells, thereby relaxing bronchial smooth muscles. While salbutamol is a rather safe drug, its adverse effects include headache, tachycardia, dizziness, and anxiety, whereas its severe side effects comprise heart attack, arrhythmia, and hypokalemia in severe overdose cases.^5^ More importantly perhaps, response to bronchodilators is very heterogeneous, particularly in children.^7^ This may be the result of a complex multifactorial interplay, including phenotypic heterogeneity of asthma (*e.g.*, allergic *vs.* non-allergic), variations in the degree of lung functional abnormalities, disparities in drug deposition and therapy compliance.

Pharmacometabolomics harnesses the principles of metabolomics to therapeutics in order to elucidate the underlying biological mechanisms of action and provide insights into an individual’s drug responsiveness.^8^^,^^9^^,^^10^^,^^11^ Thus, pharmacometabolomics is a perfectly suited approach for disentangling such complexity and to stratify patients’ response to the battery of inhaled medications available to treat asthma.^12^^,^^13^^,^^14^ One common feature to all previous pharmacometabolomic studies on the response to inhaled medications in patients with asthma, is that it is based on blood or urine samples.^15^^,^^16^^,^^17^^,^^18^^,^^19^ While blood provides a systemic view at the metabolic level, exhaled breath is a largely unexplored matrix which is likely an even better proxy to assess rapid metabolic changes in the respiratory system.^20^^,^^21^^,^^22^^,^^23^^,^^24^^,^^25^ Thus, because exhaled breath contains metabolic information stemming from blood/gas exchange, but also from the respiratory system itself, it is an ideal approach to gain insights at the molecular level of response to medications targeting the lungs.

Several metabolites and entire metabolic pathways have been described to be associated with the pathophysiological mechanism of salbutamol,^5^ while its main metabolites salbutamol-4-*O*-sulfate and other conjugated metabolites can be detected in urine,^26^^,^^27^ some of its related metabolites have been previously detected in exhaled breath.^28^ Here, we followed up on this previous knowledge to test the following hypotheses: i) whether such drug-related metabolic profiles could be measured in exhaled breath of a real-world hospital pediatric population; ii) whether these are related to clinical bronchodilator response and iii) whether there are variations across patients of such pharmacometabolomic response patterns.

## Results

### Integration of real-time breath metabolomics in routine outpatient asthma consultation

**Figure 1:**
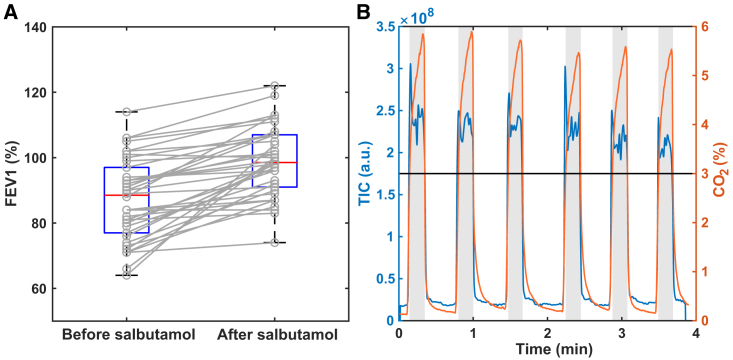
Response to inhaled medication and parallel integration of real-time breath analysis (A) FEV_1_ (%) was significantly (paired-sample t-test, df = 37, p = 7x10^−9^) increased after salbutamol inhalation. The boxplots represent the median (red line), interquartile range (blue line), and maximum and minimum values (black line) of the corresponding group of data. Stick plots show an overall increasing trend, but notably, some patients did not experience any sizable increase in lung capacity (i.e., non-responders). More information is provided in Data S1 (B) Example of the real-time readout of breath test. Real-time total ion current (TIC, in blue) detected by the mass spectrometer increases during the replicate exhalations of one patient. The exhalation maneuver is guided by a capnograph (CO_2_ time trace in orange). The gray areas correspond to the end-tidal exhalation windows (i.e., CO_2_ > 3%) used to average the mass spectral fingerprints of the patients. Please note that the timescale of the test is in the order of a few minutes, which enables a seamless integration into the clinical workflow.

In this study, we recruited *n* = 34 pediatric patients aged 6–18 years referred to the pulmonology department of the University Children’s Hospital Basel (UKBB, Switzerland) with suspected asthma, and children with confirmed asthma diagnosis seen for a regular follow-up visit. Diagnosis of asthma was according to the European Respiratory Society (ERS) clinical practice guidelines in children^1^ and based on the ERS technical standard document defining a significant bronchodilator (salbutamol) response by a change in forced expiratory volume in the first second (FEV_1_) of at least 10%.^29^ The main clinical characteristics of the *n* = 34 patients with asthma are presented in Table 1. There are 31 patients who had a single visit, two patients had two visits and one patient had three visits, resulting in a total of *N* = 38 visits. While the asthma control test (ACT) score indicates generally good control of the disease, the relatively high levels of *F*_eNO_ suggest a generalized airway inflammation in the studied asthmatic population. In addition, patients responded adequately to the medication as evidenced by the significant FEV_1_ increase (p = 7x10^−9^; Figure 1A). More detailed results of lung function and *F*_eNO_ tests can be found in Data S1. The breath test was seamlessly integrated into the clinical workflow (Figure S1). One key aspect of such integration is that, similarly to *F*_eNO_ point-of-care devices, secondary electrospray ionization high-resolution mass spectrometer (SESI-HRMS) provides a real-time metabolic readout without any discomfort. Figure 1B shows a representative example of such real-time measurement, whereby it can be appreciated how the total ion current (TIC) detected by the mass spectrometer increases during the six replicate exhalations of one patient. Note how such an increase occurs simultaneously to the increase of exhaled CO_2_ concentration. The gray bands indicate the end-tidal exhalation windows (CO_2_ > 3%) used to average the mass spectra, resulting in a high-resolution mass spectral breath fingerprint per patient and visit. More details about the measurement procedures, quality control and statistical analysis methods are provided in STAR Methods.

**Table 1:** Clinic characteristics of all included patients with asthma (*n* = 34)

Age	Gender (% female)	BMI (kg/m^2^)	ACT^a^	*F*_eNO_ (ppb)	*Δ* FEV_1_ (%)
11.2 (8.3–12.6)	26.3	17.7 (16.2–20.2)	23 (17–24)	31.6 (17.0–59.1)	9.5 (5.0–21.0)

Data are presented as median (interquartile range), unless otherwise indicated. BMI: body mass index, ACT: asthma control test, *Δ* FEV_1:_ change of forced expiratory volume in the first second comparing before and after salbutamol inhalation. *F*_eNO_: fractional exhaled nitric oxide.

a *n* = 31. More detailed patient information can be found in Data S1.

### Salbutamol induces significant metabolic changes detectable in exhaled breath

**Figure 2:**
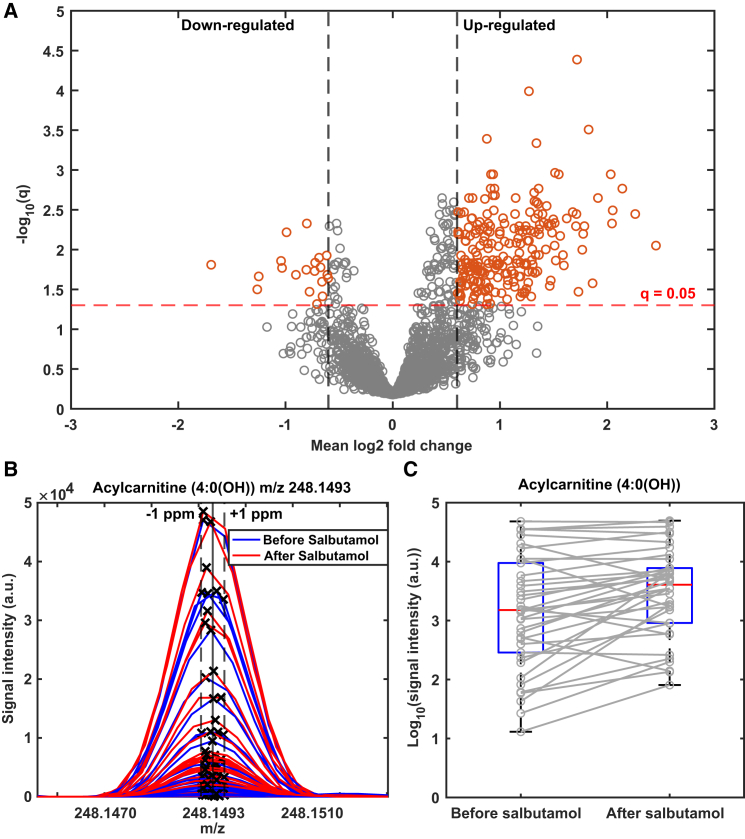
Untargeted real-time breath metabolomics reveals broad metabolic changes after salbutamol inhalation (A) Volcano plot analysis: a majority of the signal changing significantly (according to the one-sample t-test of the Log2 fold change) after salbutamol inhalation tends to increase (223 up-vs. 20 down-regulated, more information is provided in Data S2). (B) All mass spectral profiles measured in this study in the m/z region of protonated acylcarnitine (4:0(OH)). Mass accuracies within 1 ppm allow for high-confidence molecular formula assignments. Visual inspection suggests a generalized signal increase after salbutamol inhalation. (C) Signal intensity of tentatively identified acylcarnitine (4:0(OH)) shows an overall significant increase (one sample t-test of its Log2 fold change, df = 37, q = 0.007) after salbutamol inhalation. However, high patient heterogeneity can also be observed. The boxplots visualize the datasets' distribution by showing the median (red line), interquartile range (IQR; blue box) and the whiskers extending to 1.5 times the IQR. Please note the Log10 scale.

We hypothesized that changes in metabolite levels would accompany the drug action leading to a significant -as measured by FEV_1_- opening of the airways. A one-sample t-test of the Log2 fold change (Log2FC) revealed that this was the case, whereby the signal intensity of 333 mass spectral features was significantly (q < 0.05; Figure S2) altered after salbutamol inhalation. To gain further insights into whether the signals tended to increase or decrease after the drug inhalation, and the strength of such changes, the data was represented as a volcano plot (Figure 2A). A total of 223/20 features were found to be up/down-regulated (*i.e.*, abs(Log2FC) > 0.6 and q < 0.05), detailed information on these features is listed in Data S2. Figure 2B shows a representative upregulated mass spectral feature, whereby it can be appreciated that the overall signal intensity tends to increase upon drug administration. The very high mass accuracy achieved −well below ±1 ppm− after calibration in our computational pipeline, enables nearly unambiguous matching to a molecular formula of our mass spectral features, which in turn allows for mapping such formulae against metabolite databases. As a result, 63 features had a hit in at least one of the databases queried (Data S3). Thus, for example, the peak illustrated in Figure 2B, could be mapped to Acylcarnitine (4:0(OH)). Figure 2C shows how the signal of Acylcarnitine (4:0(OH)) behaves before and after salbutamol inhalation for each patient. Similar to FEV_1_ behavior (Figure 1A), most patients show a significant increase (q = 0.007).

### Biochemical interpretation of observed metabolic changes

**Figure 3:**
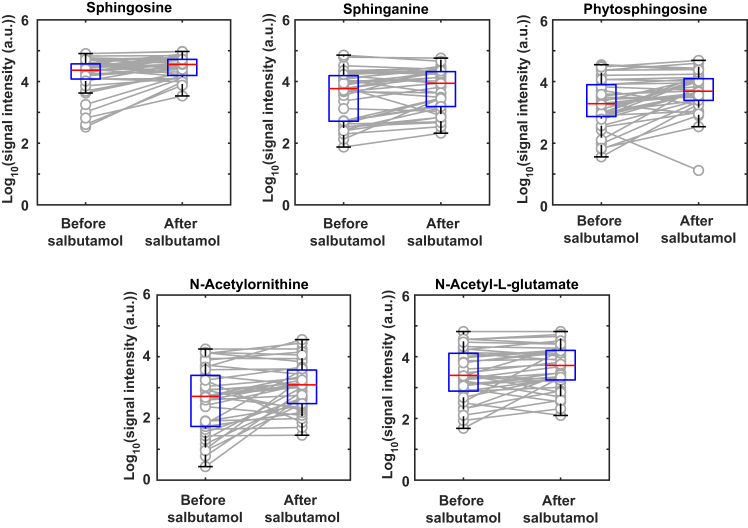
Significant upregulation of sphingolipid metabolism (top) and arginine biosynthesis (bottom) metabolites are associated with salbutamol inhalation Signal intensity of the compounds flagged by pathway analysis shows an overall significant increase in signal intensity (one sample t-test of its Log2 fold change data, df = 37, q = 2 × 10^−^^4^, 0.002, 2 × 10^−^^4^, 0.01 and 0.04, respectively from left to right). However, some individuals show an opposite trend to the general population. Please note the Log10 scale. The black error bars of the boxplot are the 95% confidence interval, the bottom and top of the blue box are the 25th and 75th percentiles, the red line inside the box is the median, and any open circles that are outside the error bars are the outliers. More information is available in Figure S3, Table S1 and Data S3.

To allow for a more in-depth interpretation of the observed metabolic changes upon salbutamol inhalation, we conducted an enrichment analysis.^30^ The analysis suggested that sphingolipid metabolism (*p* = 0.044) and arginine biosynthesis (*p* = 0.013) is significantly altered as a result of the bronchodilation (Figure S3 and Table S1). Responsible metabolites contributing to the sphingolipid metabolism pathway were sphingosine, sphinganine (*i.e.*, dihydrosphingosine), and phytosphingosine, which were all increased after salbutamol inhalation (mean Log2FC = 0.91, 0.72 and 1.37, q = 2 × 10^−^^4^, 0.002 and 2 × 10^−^^4^, respectively). On the arginine biosynthesis pathway, the detected mass spectral features were mapped to a total of six compounds, whereby two of them were significantly increased: N-Acetylornithine and N-Acetyl-L-glutamate (mean Log2FC = 1.52 and 0.75, q = 0.01 and 0.04, respectively). Figure 3 shows how these exhaled metabolites were indeed overall systematically increased upon inhaling the medication.

**Figure 4:**
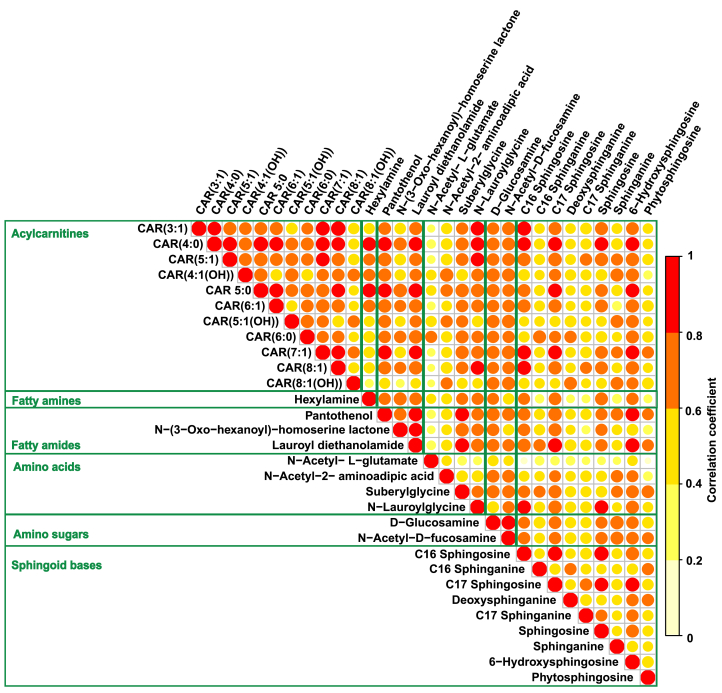
Altered metabolite classes associated with salbutamol therapy Correlation heatmap of six metabolite families was found to correlate with significant hits flagged by pathway analysis. Each dot represents the correlation coefficient of corresponding features, and the color bar represents the range of correlation coefficient values from 0 (light yellow) to 1 (red). CAR(m:n) represents carnitine and acyl group (m:n), whereby m is the chain length of the acyl group and n is the number of double bonds; (OH): hydroxy group substituents in acyl group. More compound information can be found in Data S3.

Enrichment analysis based on the KEGG database only provides limited metabolite coverage. For that reason, to leverage further evidence on the altered pathways, we extended our search to related metabolites in additional metabolite databases (*i.e.*, RefMet) and determined whether they correlated with the five compounds from Figure 3. Please note that, therefore, the identification confidence level of these compounds was based on the matching with metabolite databases using molecular formulae (*i.e.*, level 4 according to Schymanski et al.^31^). However, it should also be noted that the sphingoid bases of the sphingolipid metabolism pathway have been previously identified in exhaled breath condensate from healthy subjects at an identification confidence level 2a (*i.e.*, probable structure by library spectrum match).^32^ After excluding exogenous compounds, an additional 26 metabolites −with absolute log2(FC) ≥ 0.6 and r ≥ 0.6− could be mapped to fatty acyl (including acylcarnitines), amino acid, and sphingoid base metabolism. Figure 4 shows the resulting correlation matrix of these 26 metabolites, together with 4 significant hits metabolites from the enrichment analysis, grouped by chemical class. All compounds showed a positive correlation, whereby intra-class correlation is particularly obvious for acylcarnitines, and fatty amides.

The most extensive group corresponds to the acylcarnitines which include 11 compounds, these acylcarnitines correspond to short-chain (acyl-groups with two to five carbons, C2-C5) and medium-chain (C6-C12). Their corresponding Log2FC values ranged from 0.61 to 1.36. From the inspection of Figure 4, it becomes apparent for the acylcarnitines the very high intra-group correlation. Furthermore, we identified four compounds belonging to the fatty acyl class together with acylcarnitines, three of which are fatty amides (lauroyl diethanolamide, N-(3-Oxo-hexanoyl)-homoserine lactone, pantothenol), and one a fatty amine (hexylamine).

Sphingoid bases were the second largest group in the network, including sphingosine, sphinganine, and phytosphingosine, which were previously flagged in the enrichment analysis, along with 6-Hydroxysphingosine, deoxysphinganine, sphingosine, and sphinganine (both C16 and C17). The third largest group associated with the arginine biosynthesis pathway included the previously flagged N-Acetyl-L-glutamate, along with N-acetyl-2-aminoadipic acid, suberylglycine, and N-lauroylglycine. Finally, we identified two amino sugars (D-glucosamine and N-Acetyl-D-fucosamine), which showed good correlations with other groups, especially acylcarnitines.

### Clustering of responders vs. non-responders reveals patient heterogeneity

Finally, we aimed to gain further insights into two subpopulations: we grouped our data as responders (*Δ* FEV_1_ ≥ 10%, *n* = 17) and non-responders (*Δ* FEV_1_ < 10%, *n* = 17). Indeed, it was confirmed that both subgroups showed a significant (*p* < 0.01) different response to the medication (as assessed by *Δ* FEV_1_; Table S2). In contrast, no significant association was found for *F*_eNO_ inflammation marker concentrations (*p* = 0.78). At the breath metabolic level, we initially ran a two-sample t-test to identify differences between both groups. The histogram of *p*-values stemming from such univariate analysis of the 2,394 mass spectral features shows a non-flat distribution, indicating initially that there might be indeed significant differences between responders and non-responders (Figure S4). However, after adjustment for false discovery, no features remained significant (*i.e.*, q < 0.05), suggesting that our study is underpowered for such univariate subgrouping analysis.

**Figure 5:**
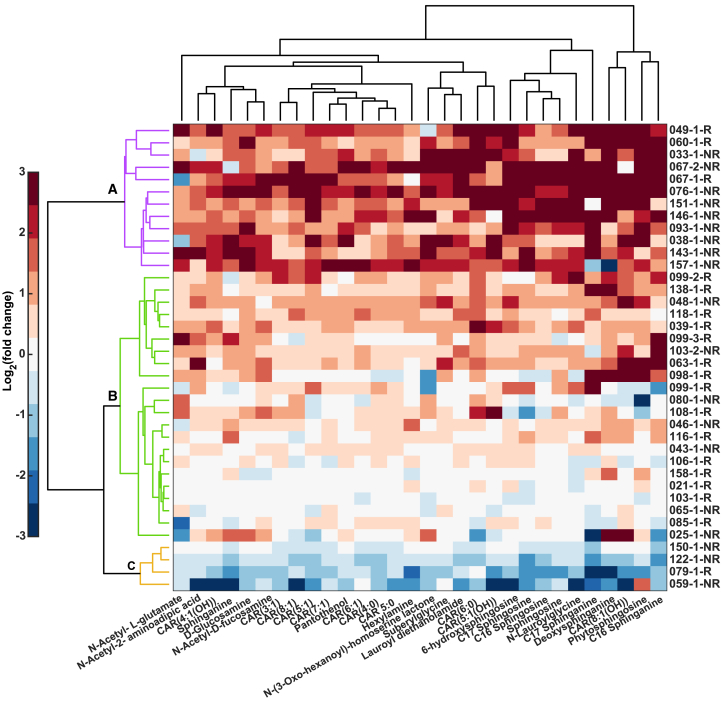
Heterogeneity of response to medication and its association with exhaled metabolites is revealed by characteristic patients’ metabotypes Cluster analysis of regulated compounds from Figure 4 for all patients. Patient coding: Patient ID-Visit number-Responder or non-responder to salbutamol. The color bar represents the range of scaled mean Log2 fold change data from −3 (navy blue) to 3 (deep red). Custer A is dominated by non-responders (75% NR vs. 25% R) with an upregulated trend of the metabolites after salbutamol inhalation; cluster B comprises the majority of the responders (32% NR vs. 68% R) whereby moderate changes in signal intensities of the metabolites between before and after salbutamol are observed; cluster C is a small cluster of four patients, again dominated by non-responders (75% NR vs. 25% R), but in contrast to cluster A, these patients display a trend toward the down-regulation of exhaled metabolites after inhaled therapy. A significant (chi-square test, *p* = 0.03) association between cluster and response to medication was observed. More responders and non-responders information can be found in Table S2 and Data S1.

We then focused our analysis on the altered metabolic pathways and metabolites identified in Figure 4. Figure 5 displays the result of a hierarchical cluster analysis. Three main patients’ clusters emerged from this analysis: cluster A (purple) comprises 12 patients and is dominated by non-responders (3 responders vs. 9 non-responders). In contrast, cluster B (green) is dominated by responders (15 responders vs. 7 non-responders). Finally, cluster C comprises a small subset of patients 1 responder and 3 non-responders. Interestingly, this small group of non-responders shows a general decrease of the signal intensity of the metabolites upon therapeutic intervention. This contrasts with the non-responders from cluster A, which show a stark increase in the exhaled metabolites. The cluster dominated by responders (cluster B) shows an overall moderate-to-no-change as a result of the intake of the bronchodilator. Overall, we observed a significant (chi-square test, *p* = 0.03) association between cluster and response to medication.

## Discussion

In this work, we show evidence that real-time breath analysis of metabolites can be integrated into routine clinical workflow, in the context of asthma control and monitoring. Pre- and post-bronchodilator lung function is at the forefront of routine clinical workflows to assess the response to medication. While a mechanical readout is key to understanding the efficacy of the medication, adding a layer of metabolic information retrieved directly from the respiratory system may help to better characterize the broad spectrum of different responses often observed in everyday clinics, especially in heterogeneous respiratory diseases such as asthma. *F*_eNO_ is an excellent example of how an exhaled cue complements the constellation of clinical characteristics and lung function tests used to assess inflammation in respiratory diseases.

Here, we present how a companion breath test −along with the standard pre- and post-bronchodilator lung function− adds a wealth of metabolic information. Along with the expected significant increase in lung volume, we observed a systematic and significant alteration of the signal intensity of hundreds of mass spectral features upon salbutamol inhalation. This is consistent with a previous study whereby 131 breath features were significantly altered after the inhalation of salbutamol, but not after the inhalation of placebo.^28^ Despite the very different study designs and mass spectrometric instrumentation used, we find an overlap (within 5 ppm) in 98 of 131 features, whereby 46 of those 98 features have the same upregulated trend. This suggests that salbutamol induces a rapid metabolic cascade, whereby compounds are building up. Indeed, our data suggests that the concentration in exhaled breath of a wide range of metabolites is increased as a result of bronchodilation due to salbutamol. However, it is difficult to disentangle whether these changes occur because of an increased local cellular metabolism or simply because new areas of the respiratory system −previously inaccessible− are recruited. To account for a possible bias due to the increased lung capacity, we ran our analysis adjusting the mass spectral signal intensities with FEV1 and yet confirmed the significance of our observations. However, because the breath test took place 45–60 min after salbutamol inhalation, it is reasonable to assume a steady state and hence further interpretations were made on volume-unadjusted data.

When attempting to further interpret the meaning of such changes, our analysis points toward an activation of the sphingolipid metabolism and arginine biosynthesis. These altered pathways are indeed very plausible, as they fit in the well-known mechanistic framework of salbutamol action mode. The inhaled β_2_-adrenoceptor agonist salbutamol binds reversibly to β_2_-receptors in the cells of the smooth muscle respiratory tract, to activate adenyl cyclase and then lead to the conversion of ATP to cAMP. Upon activation, cAMP initiates a series of intracellular biochemical reactions. These reactions ultimately lead to the suppression of bronchial smooth muscle contraction. This suppression facilitates the relaxation of the smooth muscle and induces bronchodilation, which is the observed therapeutic effect of salbutamol.^5^ The significant increase and high intra-group correlation of fatty acyls (including acylcarnitines; Figures 3 and 4) upon salbutamol inhalation, may point to an important contribution of these metabolites to provide the required energy in the form of ATP production to sustain ultimately muscle relaxation.^33^ Our data also points toward activation of sphingolipids metabolism after salbutamol intake. For example, we observed a significant increase in sphingosine, sphinganine, and phytosphingosine (Figure 3), which are basic backbones of sphingolipids in animal tissues. This is also consistent with previous multi-omics data of asthma control with salbutamol,^34^ whereby sphingolipid metabolism was identified as modulated (e.g., sphingosine-1-phosphate pathway) in response to salbutamol treatment. The pathway analysis also pointed towards the arginine biosynthesis pathway alteration. However, in contrast with the above-mentioned sphingolipid metabolism and acylcarnitine mobilization, no direct link with the known mechanism of action of salbutamol could be established. However, this observation is also consistent with a previous breath analysis study, which identified two arginine pathways downregulated in asthmatic children as compared to healthy controls.^35^

Finally, when attempting to cluster the patients with regard to these metabolic pathways, we found a significant association with whether or not the children responded to salbutamol. Interestingly, we observed two very distinct metabotypes of non-responders. One group comprising the majority of the non-responders (cluster A in Figure 5) where the acylcarnitines, sphingosines, and related metabolites were systematically upregulated, and another smaller group (cluster C in Figure 5) showed an opposite trend. The third main cluster of patients (cluster B in Figure 5) consisted of mainly responders. The significant − albeit weak (chi-square test, *p* = 0.03) – association between metabotypes and response to medication, suggests that this technique may be useful in future studies to investigate metabolomic variations in bronchodilator response in children and relate these to clinical outcomes and asthma control. We hypothesize that further development of this technique may not only support the therapeutic management of asthma but could potentially be leveraged by the pharmaceutical industry to further refine ongoing clinical trials on developing inhaled medications.

### Limitations of the study

While these observations are consistent with prior literature and the known mechanism of action of salbutamol, we should also acknowledge the important limitations of this study: while the studied population is highly representative of a real-world pediatric asthma outpatient clinic, the sample size is certainly very limited. Also, while the mass accuracy achieved in this study allows for unambiguous molecular formula assignment, compound identification is based solely on that (*i.e.*, identification confidence level 4^31^). Validation in an independent cohort and unambiguous structural elucidation using chemical standards^36^ are pending.

## Resource availability

### Lead contact

Further information and requests for resources and information should be directed to and will be fulfilled by Pablo Sinues (pablo.sinues@unibas.ch).

### Materials availability

This study did not generate new unique materials.

### Data and code availability

• The RAW files of the real-time breath measurements are available from the repository Mendeley Data: https://doi.org/10.17632/rtv6mpf3kb.2.
• This article does not report the original code.
• Any additional information required to reanalyze the data reported in this article is available from the lead contact upon request.

## Acknowledgments

PS received funding from 10.13039/501100011318Fondation Botnar (Switzerland) and 10.13039/501100001711Swiss National Science Foundation (PCEGP3_181300). We thank Isabel Gonzalez Novoa and Stefanie Hammer for conducting the interviews and document filling of patients’ data. Mélina Richard is gratefully acknowledged for coordinating the study.

## Author contributions

Conceptualization, P.S., J.U., and U. F.; methodology, P.S., A.J., and J.U.; investigation, J.Z.; formal analysis, J.Z. and K.D.S. writing – original draft, J.Z.; writing – review and editing, P.S., J.U., K.D.S., D.T., and U. F.; funding acquisition, P. S.; resources, J.U. and A.J.; supervision, P.S. and U.F.

## Declaration of interests

P.S. is a cofounder of Deep Breath Intelligence AG (Switzerland), which develops breath-based diagnostic tools. K.D.S. is a part-time employee of Deep Breath Intelligence AG (Switzerland). All other authors declare no competing interests. The data-processing pipeline discussed here has been incorporated in the European patent 21185400.5, filed on July 13^th^, 2021.

## STAR★Methods

### Key resources table

**Table:** 

REAGENT or RESOURCE	SOURCE	IDENTIFIER
**Chemicals, peptides, and recombinant proteins**		

Salbutamol	GlaxoSmithKline, United Kingdom	Ventolin, Salbutamol 100 μg/dose, https://public.gsk.co.uk/products/ventolin.html
α-Terpinene (C_10_H_16_, 100 ppb)	Dalian Special Gases, China	Customized standard gas, http://www.dl-gas.com
Mass spectrometry calibration solution	Thermo Fisher Scientific, Germany	Pierce™ FlexMix™ Calibration Solution, https://www.thermofisher.com/order/catalog/product/A39239
0.1% formic acid in water (Primary solution)	Sigma-aldrich, Merck, USA	0.1% Formic Acid Solution https://www.sigmaaldrich.com/CH/en/product/mm/159013?srsltid=AfmBOorFvdTwjZRZ-ZU99uHQj8BpKPJKSDHOdtCWRDiwPsU6ShWkO2Sw
CO_2_ standard gas (5%)	Garbagas, Switzerland	Customized 5% CO2 standard gas https://www.carbagas.ch/de/search/gas

**Deposited data**		

Raw data	Mendeley Data: https://data.mendeley.com/	https://doi.org/10.17632/rtv6mpf3kb.2

**Software and algorithms**		

MATLAB version 2022a	MathWorks Inc., USA	https://ch.mathworks.com
Thermo Exactive Plus Tune software version 2.9	Thermo Fisher Scientific, Germany	https://www.thermofisher.com
RawFileReader version 5.0.0.38	Thermo Fisher Scientific, Germany	https://www.thermofisher.com
MetaboAnalyst version 6.0	MetaboAnalyst	https://www.metaboanalyst.ca/home.xhtml; RRID: SCR_015539
MetaboAnalystR version 3.0.3	MetaboAnalyst	https://www.metaboanalyst.ca/; RRID: SCR_015539
R version 4.3.2	The Comprehensive R Archive Network (CRAN)	https://cran.r-project.org; RRID: SCR_001905

**Other**		

Clinical trial information	Exhaled Breath Analysis by Secondary Electrospray Ionization - Mass Spectrometry in Children and Adolescents (EBECA)	ClinicalTrials.gov ID NCT04461821 https://clinicaltrials.gov/study/NCT04461821?term=pablo%20sinues&rank=1. This is approved by the Ethics Committee of North–Western and Central Switzerland (ID 2020-00778)
Secondary electrospray ionization source	Fossil Ion Tech, Spain	SUPER SESI, https://www.fossiliontech.com/
High-resolution mass spectrometer	Thermo Fisher Scientific, Germany	Q Exactive Plus, https://www.thermofisher.com
Lung function test machine	Vyaire Medical, U.S.A.	Vyntus™ BODY Plethysmograph, https://www.vyaire.com/products/vyntus-body-plethysmograph
FeNO analyzer	ECO Medics, Switzerland	ANALYZER CLD 88 sp FeNO analyzer, https://www.ecomedics.com/products/analyzer-cld-88-sp/
Exhalion (CO_2_ and flowrate monitor)	Fossil Ion Tech, Spain	Exhalion, https://www.fossiliontech.com/exhalion
Electrospray capillary emitter (20-μm ID)	Fossil Ion Tech, Spain	20-μm ID https://www.fossiliontech.com/nanoesi-emitters
Electrospray capillary emitter (50-μm ID)	Fossil Ion Tech, Spain	50-μm ID https://www.fossiliontech.com/nanoesi-emitters

### Experimental model and subject details

#### Study design and patients recruitment

In this pre-post study, we recruited children aged 6–18 years referred to the pulmonology department of the UKBB with suspected asthma, and children with confirmed asthma diagnosis seen for a regular follow-up visit. Diagnosis of asthma was according to the ERS clinical practice guidelines in children^1^ and based on ERS technical standard document defining a significant bronchodilator response (salbutamol) by a change in FEV_1_ of at least 10%.^29^ We recruited a total of 62 patients from the outpatient clinic of UKBB. After excluding children without asthma, and dropouts due to technical difficulties during lung function or breath analysis, 34 patients with confirmed asthma were included for the final analysis (some patients with confirmed asthma diagnosis were also recruited although their *Δ*FEV_1_ results were lower than 10% at the time when they joined our experiment). More details can be found in Figure S5 study consort diagram. There are 31 patients had a single visit, two patients had two visits and one patient had three visits, resulting in a total of *N* = 38 visits. These 38 samples/visits were further grouped as responders (*Δ*FEV_1_ ≥ 10%; 19 samples) and non-responders ((*Δ*FEV_1_ < 10%; 19 samples). Detailed information of patients’ gender, age, BMI, lung function test, and *F*_*eNO*_ test results can be found in Table 1 and Data S1, patients’ ancestry, race, and ethnicity are not available in this study. Two-sample t-tests (df = 37, *p* > 0.05) indicated there are no significant differences between male and female patients with those regulated metabolites we detected in patients’ breath data. The study was approved by the Ethics Committee of North–Western and Central Switzerland (ID 2020-00778). All subjects gave written, informed consent to participate in advance, all the measurements were conducted following rules of the Declaration of Helsinki.

### Method details

#### Instruments and measurements

Each patient provided two sets of real-time breath measurements (Figure S1): a baseline breath analysis measurement was performed 30 min before a baseline lung function and *F*_eNO_ test. In addition, an interview of the asthma control test (ACT)^37^ with a score ranging from 0 = no asthma control to 25 = perfect asthma control was done, and patient’s medical condition was assessed at the time of the baseline breath measurement. Subsequently, salbutamol (400 μg; Ventolin 100 μg/dose, GlaxoSmithKline, United Kingdom) was inhaled. Thereafter, the patients underwent a follow-up lung function test and a further clinical consultation with the doctor to assess the lung function (Vyntus BODY Plethysmograph, Vyaire Medical, U.S.A.) and *F*_eNO_ (ANALYZER CLD 88 sp FeNO analyzer, ECO Medics, Switzerland) tests, which was then followed by the second follow-up breath measurement. The whole process between the baseline and follow-up breath analyses took around 1.5 h, and time between salbutamol inhalation and follow-up breath measurement was around 1 h. Patients were asked to avoid eating, drinking (except water), cosmetics and brushing teeth at least 1 h prior to the breath analysis measurements.

The real-time breath analysis instrumentation consisted of a secondary electrospray ionization-high resolution mass spectrometry (SESI-HRMS; SuperSESI, Fossil Ion Tech, Spain; Q-Exactive Plus, Thermo Fisher Scientific, Germany) platform.^38^^,^^39^^,^^40^ Q Exactive Tune software (version 2.9) was used to control the mass spectrometry (MS) for real-time breath measurements. Each breath measurement set consisted of six replicate exhalations measured in positive and negative ion mode, with full-scan mode over a range from m/z 70–1000 under the resolution of 140,000. The temperature of ion transfer tube was 275°C and S-lens RF level of 55. The automatic gain control (AGC) target of the MS was set to 10^6^, maximum injection time was 500 ms and microscans value was 2. The MS was calibrated monthly by used the calibration solution (Pierce FlexMix Calibration Solution, Thermo Fisher Scientific, Germany) A 20-μm ID silica capillary emitter coupled with a 50-μm ID silica capillary (Fossil Ion Tech, Spain) were used for the electrospray formation, the primary solution was the 0.1% formic acid in water (0.1% Formic Acid Solution, Sigma-aldrich, Merck, USA). Detailed SESI source settings are as follow: sample line temperature 130°C, ion chamber temperature 90°C, solvent reservoir pressure 1.3 bar, sheath gas value 60, auxiliary gas value 2, spray voltage 3.5 kV, exhaust mass flow controller 0.7 L/min, and nitrogen mass flow through the source was 0.4 L/min, to allow 0.3 L/min of breath sample entering the ionizer. Exhalation maneuvers were standardized by biofeedback (CO_2_ percentage, flow rate and exhaled volume) provided a capnograph and flowmeter (Exhalion; Fossil Ion Tech, Spain), and this Exhalion instrument was calibrated by 5% CO_2_ (Garbagas, Switzerland) weekly.

#### Quality control

Before the first patient breath measurement of the day, a quality control (QC) measurement of standard gas (α-Terpinene, 100 ppb, Dalian Special Gases, China) was conducted with SESI-HRMS. Measured signal intensity of gas standard was compared with previous collected QC database following the Nelson control rules^41^ in the self-developed MATLAB-app (Figure S6). When the comparison result complies the control rules, the app will display a green light as a ‘go’ signal indicates the SESI-HRMS platform is ready to measure breath samples, otherwise it will show a red light as ‘no-go’ signal suggesting the platform is not-ready and needs to be tune. Besides, the MATLAB-app will compare the background level between the current QC measurement and previous measurements in the database to check if any potential contamination in the instruments. During the period of all the measurements with patients in this study (from November 2020 until November 2022), the SESI-HRMS platform shows a coefficient of variation of 20.1% with the RAW signal intensities of QC measurements (Figure S7).

### Quantification and statistical analysis

#### Statistical analysis & biological interpretation

Pre-processing of exhaled breath MS raw files was performed using our patented data processing pipeline (European patent No. 20186274.5 and 21185400.5).^40^ Briefly, the acquired RAW files were preprocessed using in-house C# console apps based on Thermo Fisher Scientific’s RawFileReader (version 5.0.0.38) and MATLAB (version R2022a; MathWorks Inc., USA). A representative mass spectrum per patient was obtained by averaging the scans during the replicate exhalation windows whereby CO_2_ concentrations were >3%. Thus, ensuring that only the end-tidal fraction was considered in further analyses. Averaged centroid and profile mass spectra were subsequently calibrated to achieve a mass accuracy within ±1 ppm, which allowed for unambiguous molecular formulae assignments. Subsequent apodization of artifact satellite peaks^42^ was accomplished. Finally, the centroid dataset was binned ±1 ppm using MATLAB’s *ksdensity* function. This resulted in a feature list of size 4641 in positive mode and 1842 in negative mode. Afterward during postprocessing, the sparsity of the 6483 x 76 (features x samples) data matrix was reduced by considering the features present in at least 60% of the samples of after salbutamol intake measurements. This reduced the matrix dimensionality to 2394 x 76. Zeros were then imputed using regression on order statistics function in R.^43^^,^^44^ Finally, in order to compute the changes before and after salbutamol and for the data normalization as well, we computed the Log2 fold change (Log2FC) per subject by taking the Log2 of the ratio of the breath measurement after over before salbutamol inhalation. The resulting 2394 x 38 matrix was subjected to a one sample t-test (df = 37) and *p*-values were adjusted (i.e., *q*-values) for multiple comparison using Storey’s.^45^ Associations between clusters and responsiveness to medication was accomplished via Pearson’s chi-squared test of independence at a significance level of 5%. Further data interpretation was accomplished using pathway enrichment based on mummichog algorithm^46^ with MetaboAnalystR package.^30^ The allowed mass tolerance was 3 ppm, with ion adduct forms of [M + H]^+^, [M-H]^-^, [M(^13^C)+H]^+^, [M(^13^C)-H]^-^ and selecting significant differential peaks the top 10% of the input list and using KEGG database for the mapping of metabolites. Additionally, customized R codes based on MetaboAnalystR package^47^ was used for metabolite mapping of accurate masses in the database of RefMet: A Reference list of Metabolite names.^48^ Then matched formulas and compound names were further cross-referenced with Human Metabolome Database (HMDB), PubChem and METLIN. Two-sample t-test was applied to investigate the difference of Log2 fold change data between responders and non-responders, the resulted *p*-values were adjusted (i.e., *q*-values) for multiple comparison using Storey’s.

### Additional resources

The study was part of the study of Exhaled Breath Analysis by Secondary Electrospray Ionization - Mass Spectrometry in Children and Adolescents (EBECA), ClinicalTrials.gov ID NCT04461821. This is approved by the Ethics Committee of North–Western and Central Switzerland (ID 2020-00778). More information can be found at:

https://clinicaltrials.gov/study/NCT04461821?term=pablo%20sinues&rank=1.

## Supplemental information

Document S1. Figures S1–S7, Tables S1 and S2

Data S1. Sample Information, lung function test, and FeNO test results, related to Table 1

Data S2. information of up-regulated and down-regulated breath features after salbutamol inhaled, related to Figure 2

Data S3. identified compounds list, related to Figure 3, Figures 4 and 5
