# Supplementary material for: Pharmacometabolomics via real-time breath analysis captures metabotypes of asthmatic children associated with salbutamol responsiveness
Source: iScience. 2024 Nov 20;27(12):111446. doi: 10.1016/j.isci.2024.111446 (PMC11652886; doi:10.1016/j.isci.2024.111446)
Supplement: Document S1. Figures S1–S7, Tables S1 and S2 [file mmc1.pdf]

iScience, Volume *27*

Supplemental information

Pharmacometabolomics via real-time breath analysis captures metabotypes of asthmatic children associated with salbutamol responsiveness

Jiafa Zeng, Jakob Usemann, Kapil Dev Singh, Anja Jochmann, Daniel Trachsel, Urs Frey, and Pablo Sinues

Supplementary Material

Table of content

Figure S1. Experimental design diagram.

Figure S2. Histogram plot of p and q values from one sample t-test of before and after salbutamol intake, related to Figure 2a.

Figure S3. Enrichment analysis plot. Related to Figure 3 and Table S1.

Figure S4. Histogram plot of p and q values from two sample t-test of responder and non-responder groups

Figure S5. Study consort diagram

Figure S6. Screenshot of inhouse MATLAB-app.

Figure S7. Signal intensities of gas standard used for quality control during the period of all breath measurements (from November 2020 to November 2022).

Table S1. Pathway information of enrichment analysis

Table S2. Clinical characteristics of responders and non-responders

Reference

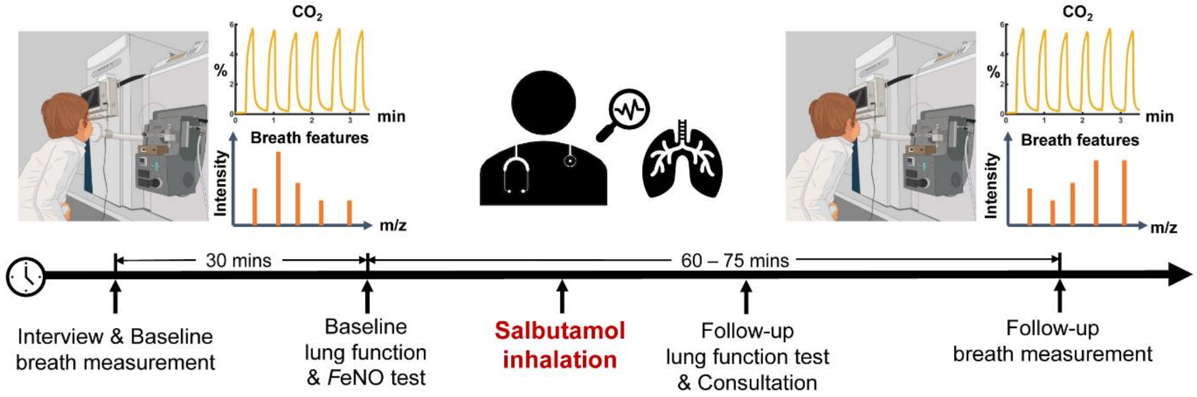

Figure S1. Experimental design diagram. The baseline breath measurement, baseline lung function test and F_{eNO} were conducted before salbutamol inhalation. Subsequently, a follow-up lung function test, consultation and follow-up breath measurement were performed.

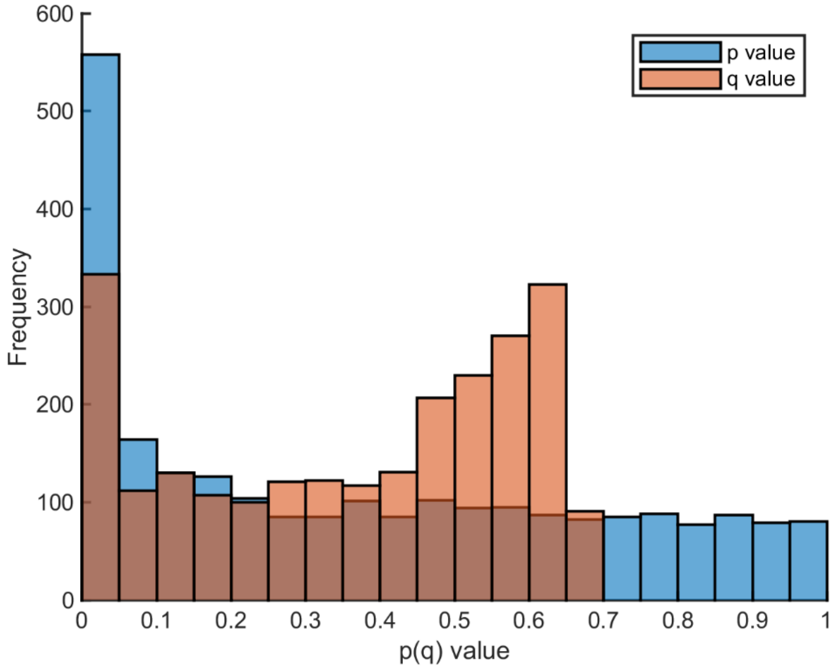

Figure S2. Histogram plot of p values from one sample t-test (blue) of log2 fold change data of before and after salbutamol inhalation breath data, and q values after positive false discovery rate correction (orange). related to Figure 2a.

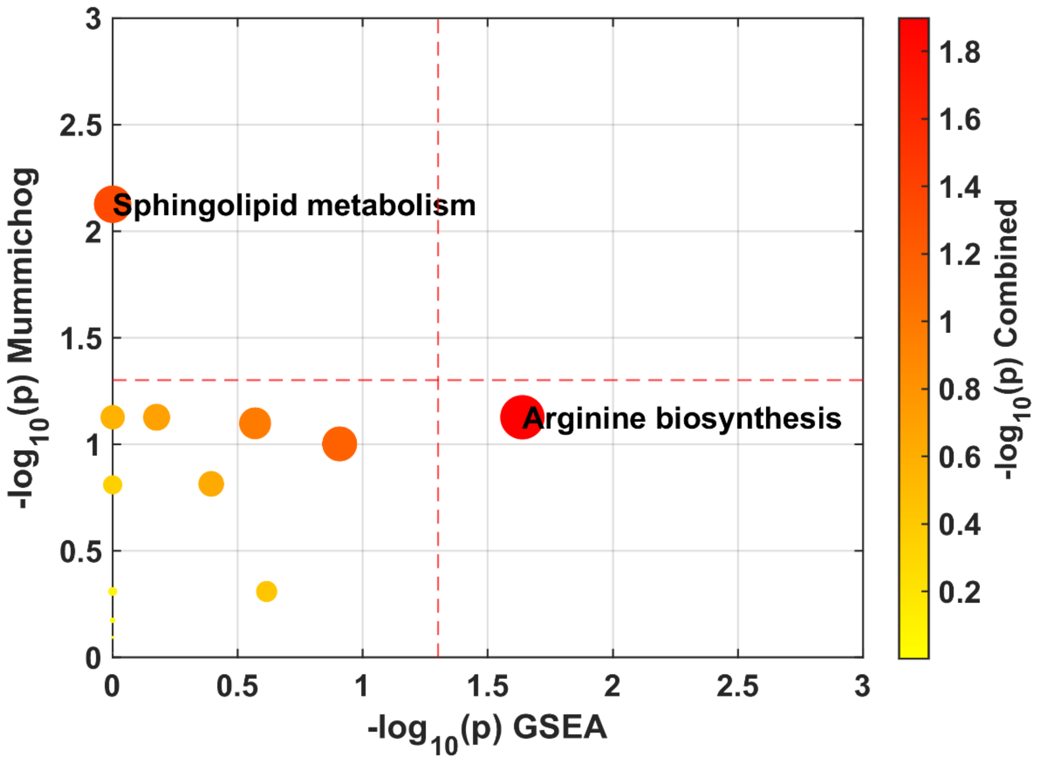

Figure S3. Enrichment analysis plot using Mummichog and GSEA algorithms. The red dash lines represent the cut-off of *p*-value of 0.05 in both axes. The color and the size represent the p value of the pathway, the bigger and the redder dot mean the lower p value, the smaller and the lighter yellow dot mean the higher p value. The color bar represents the –log10 form of p values (from 0 to 1.9; for reference $-\log_{10}(0.05) = 1.3010$, $-\log_{10}(0.01) = 2$). Related to Figure 3 and Table S1.

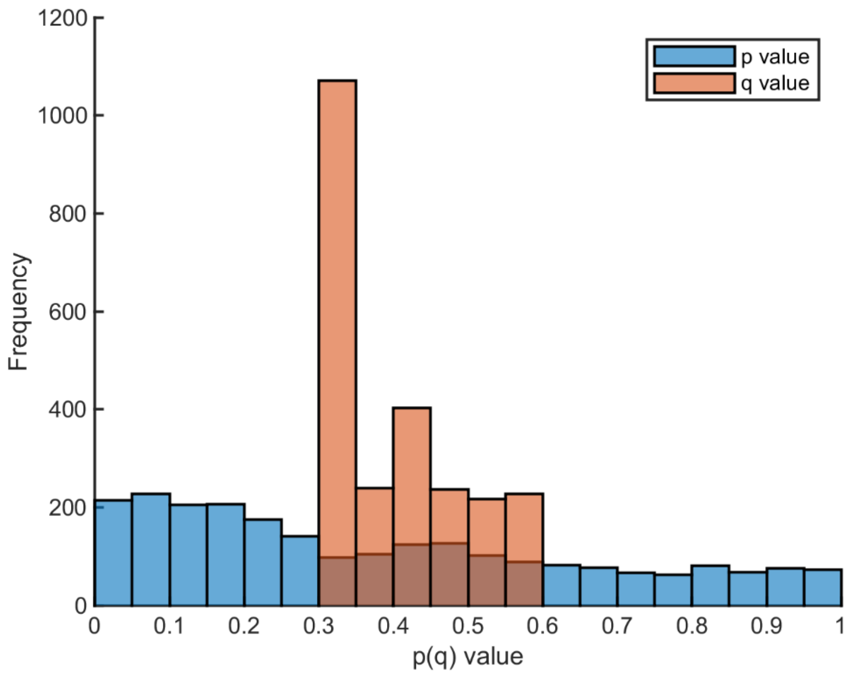

Figure S4. Histogram plot of p values (blue) stemming from a 2-sample t-test comparing responders *vs.* non-responders, and q values after positive false discovery rate correction (orange). Related to Figure 5 and Table S2.

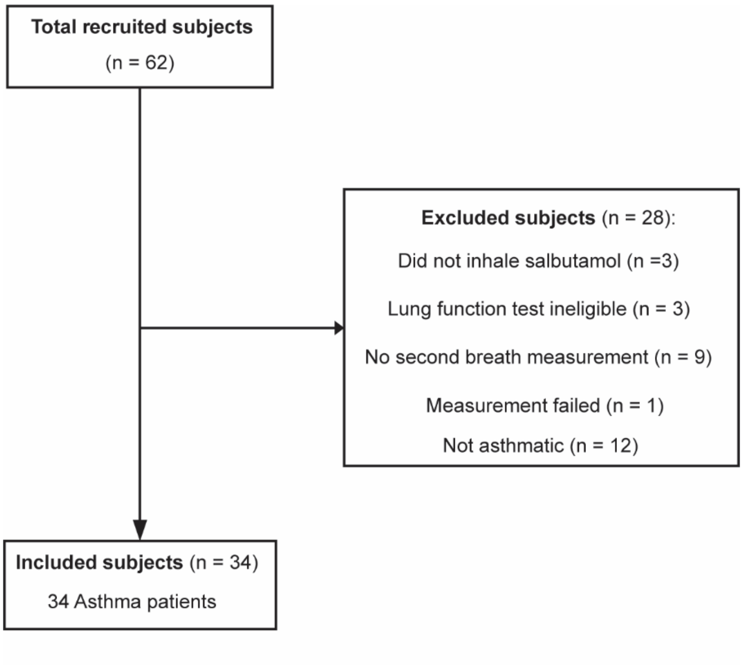

Figure S5. Study consort diagram. A total of 62 patients were initially recruited, after excluded ineligible subjects, 34 asthma patients were included in this study. Related to STAR METHODS.

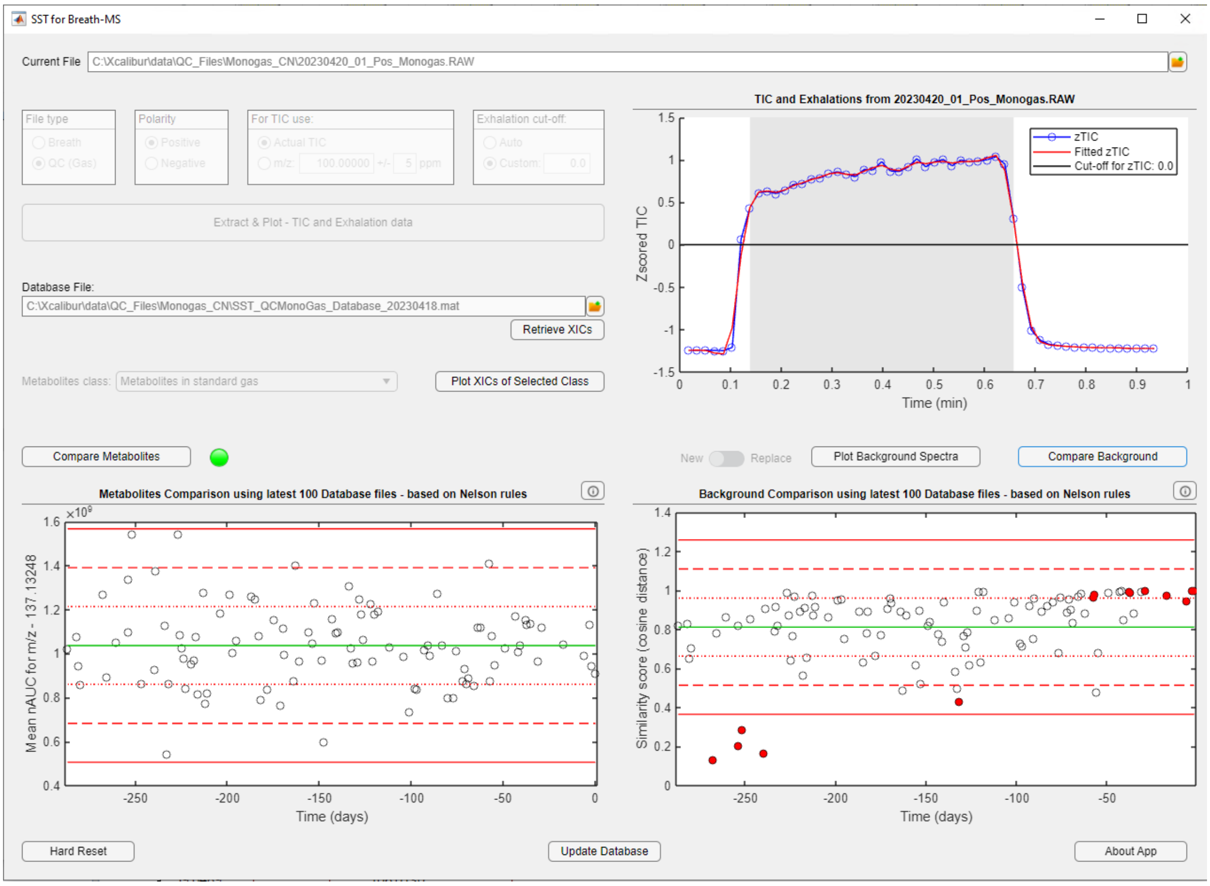

Figure S6. Screenshot of in-house MATLAB app. This app was used before every breath measurement to check instrument suitability. App provides a simple “in control” (green light) or “out-of-control” (red light) signal based

on the Nelson rules[1] using as input the current and historical signal intensities of a standard gas (α-terpinene, 100 ppb). Related to STAR METHODS.

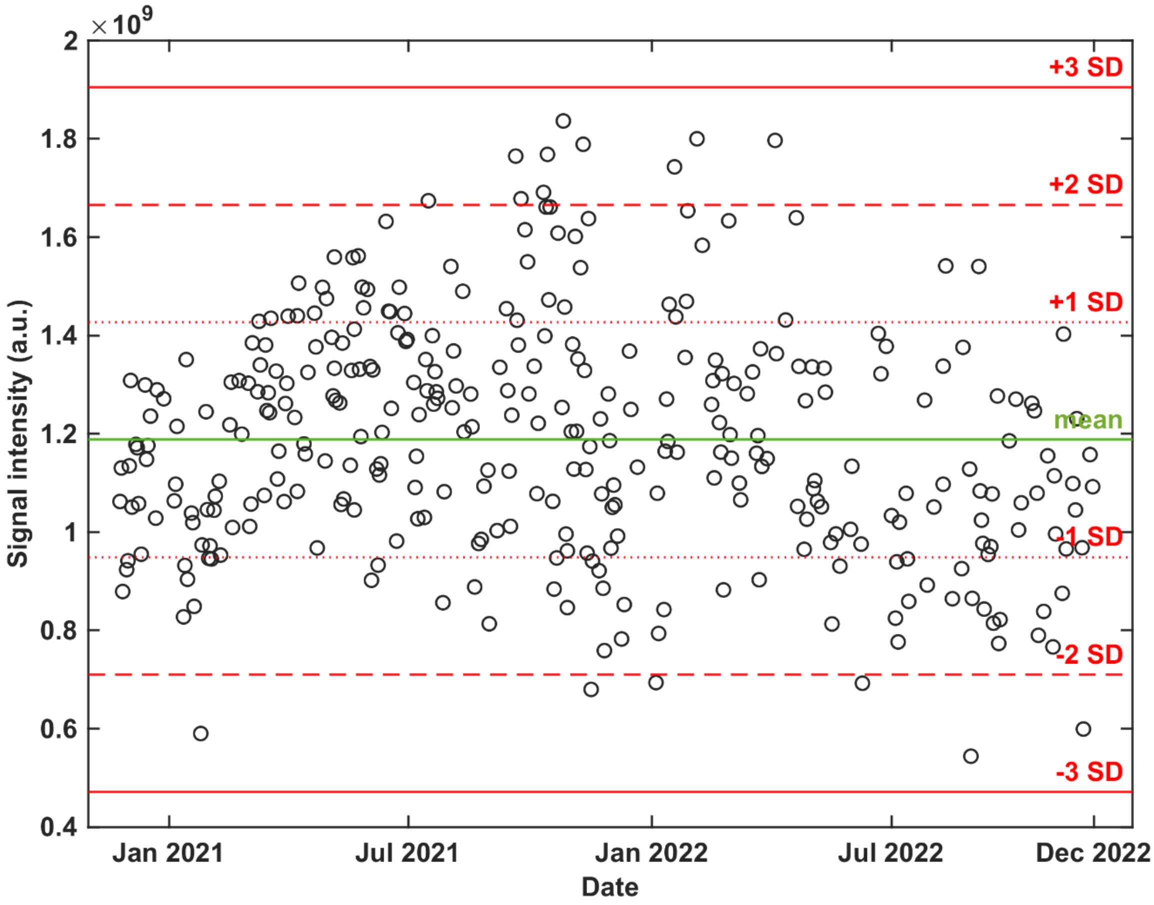

Figure S7. Signal intensities of standard gas (α-terpinene, 100 ppb) used for quality control during the period of all breath measurements (from November 2020 to November 2022). The green line means the mean of signal intensity of all datapoints, redlines means the mean +/- n* standard deviation(s) (n = 1, 2, 3). Coefficient of variation of RAW signal intensities of all measurements: 20.1%. Related to STAR METHODS.

Table S1. Pathway information of enrichment analysis. Related to Figure 3 and Figure S3.

Pathway name	Total Size	Hits	Sig Hits	*p* (Mummichog)	*p* (GSEA)	*p* (Combined)
**Sphingolipid metabolism**	**32**	**6**	**3**	**0.007**	**0.999**	**0.044**
**Arginine biosynthesis**	**14**	**6**	**2**	**0.075**	**0.023**	**0.013**
Fatty acid biosynthesis	47	6	2	0.075	0.667	0.199
Biosynthesis of unsaturated fatty acids	36	6	2	0.075	0.999	0.268
Fatty acid elongation	39	1	1	0.080	0.269	0.104
Lysine degradation	30	7	2	0.100	0.124	0.066
Fatty acid degradation	39	2	1	0.153	0.403	0.234
Amino sugar and nucleotide sugar metabolism	42	9	2	0.155	0.999	0.443
Pantothenate and CoA biosynthesis	20	8	1	0.490	0.242	0.372
Valine, leucine and isoleucine biosynthesis	8	8	1	0.490	0.999	0.840
Valine, leucine and isoleucine degradation	40	13	1	0.670	0.999	0.938
Arginine and proline metabolism	36	19	1	0.806	0.999	0.980
Tyrosine metabolism	42	34	1	0.952	0.999	0.999

Table S2. Clinical characteristics of responders and non-responders

	**Responders**	**Non-responders**	**p-value**
**Numbers**	17	17	/
**Visits**	19	19	/
**Age**	9.3 (7.1 – 12.0)	11.7 (10.2 – 14.0)	0.032
**BMI**	17.3 (15.1 – 18.5)	19.1 (17.0 – 22.7)	0.09
**Gender**	29.4% Female	29.4% Female	/
**ACT**	21(16 – 23) (n = 16)	24 (19 – 25) (n = 15)	0.15
**F_{eNO} (ppb)**	33 (23.4 – 56.3)	26.6 (12.2 – 62.1)	0.78
**Δ FEV_1 (%)**	21 (16.3 – 29)	5 (2.3 – 6)	<0.01

Data are presented as median (interquartile range), unless otherwise stated. BMI: body mass index, ACT: asthma control test, ΔFEV_1: change of forced expiratory volume in the first second comparing before and after salbutamol inhalation. F_{eNO}: fractional exhaled nitric oxide. Related to Figure 5 and Figure S4.

References

1. Nelson, L.S. (1984). The Shewhart Control Chart—Tests for Special Causes. J Qual Technol *16*, 237-239. 10.1080/00224065.1984.11978921.
